# Supplementary material for: CSF1R Ligands Expressed by Murine Gliomas Promote M-MDSCs to Suppress CD8+ T Cells in a NOS-Dependent Manner
Source: Cancers (Basel). 2024 Sep 1;16(17):3055. doi: 10.3390/cancers16173055 (PMC11394022; doi:10.3390/cancers16173055)
Supplement: Supplementary file 1 [file cancers-16-03055-s001.zip › cancers-3074102-supplementary.pdf]

Supplementary Materials

CSF1R Ligands Expressed by Murine Gliomas Promote M-MDSCs to Suppress CD8⁺ T Cells in a NOS-Dependent Manner

Gregory P. Takacs, Julia S. Garcia, Caitlyn A. Hodges, Christian J. Kreiger, Alexandra Sherman and Jeffrey K. Harrison *

Department of Pharmacology & Therapeutics, College of Medicine, University of Florida Gainesville, FL 32610, USA; juliagarcia@ufl.edu (J.S.G.); caitlyn.hodges@ufl.edu (C.A.H.)
* Correspondence: jharriso@ufl.edu

Table S1. Antibodies used for flow cytometry. Marker, fluorophore, company, catalog number and dilution factors are reported.

Cell Marker:	Fluorophore:	Company:	Catalog #:	Dilution Factor:
CD3	BV510	BioLegend	100234	1:100
CD4	FITC	BioLegend	100510	1:100
CD8	PE	BioLegend	100712	1:100
CD45	Alexa700	BioLegend	103128	1:200
CD11b	BV421	BioLegend	101251	1:100
LY6C	BV785	BioLegend	128041	1:100
LY6G	PerCP	BioLegend	127654	1:100
CD39	PE	BioLegend	143804	1:100
CD73	APC	BioLegend	127210	1:100
iNOS	APC	Invitrogen	17-5920-82	1:100
Viability Dye	Pacific Blue	Invitrogen	L34963	1:1000
FcR Block	-	BioLegend	101320	2 uL per sample

Table S2. Cytokines, antibodies, and small molecule compounds used for expansion and inhibition of MDSC differentiation or inhibition of M-MDSC suppression. Company and catalog numbers are reported.

**Cytokine/Antibody/Small Molecule**	**Company:**	**Catalog #:**
GM-CSF	R and D Systems	415-ML-020
M-CSF	R and D Systems	416-ML-010
G-CSF	R and D Systems	414-CS-005
TGF- β	R and D Systems	7666-MB-005
IFNγ	R and D Systems	485-MI-100
IL-1β	R and D Systems	401-ML-005
IL-2	R and D Systems	402-ML-020
IL-6	R and D Systems	406-ML-025
IL-12p40	R and D Systems	9807-IL-050
IL-13	R and D Systems	413-ML-005
LIF	R and D Systems	8878-LF-025
αIL-34 mAB	R and D Systems	MAB5195
αM-CSF pAB	R and D Systems	AF416-SP
αPD-L1 mAB	R and D Systems	MAB90783
αIFN-γ R2 mAB	R and D Systems	MAB773
Apocynin	R and D Systems	4663
CGS 15943	R and D Systems	1699
Celecoxib	R and D Systems	3786
Pexidartinib	R and D Systems	7590
Nor NOHA monoacetate	R and D Systems	6370
L-NMMA acetate	R and D Systems	0771
Methyl-D-tryptophan	R and D Systems	5698

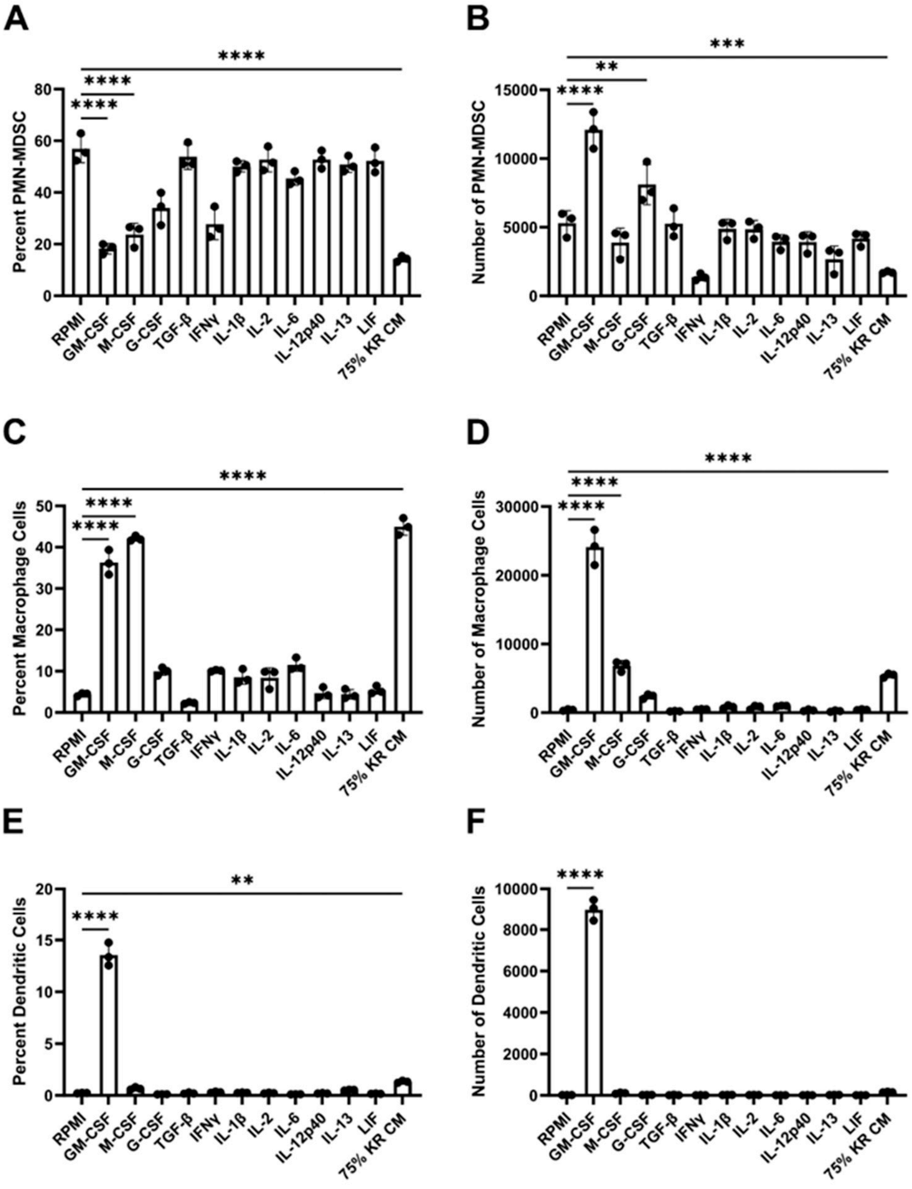

Figure S1. Glioma upregulated cytokines alter bone marrow differentiation ex vivo. Graph depicting the (**A**) percent and (**B**) numbers of Ly6C/Ly6G PMN-MDSCs generated from whole bone marrow cultured for 3 days in the presence of 40ng/mL cytokines or 75% KR158B conditioned media (n=3). Graph depicting the (**C**) percent and (**D**) numbers of F4/80 macrophages generated from whole bone marrow cultured for 3 days in the presence of 40ng/mL cytokines or 75% KR158B conditioned media (n=3). Graph depicting the (**E**) percent and (**F**) numbers of CD11c dendritic cells generated from whole bone marrow cultured for 3 days in the presence of 40ng/mL cytokines or 75% KR158B conditioned media (n=3). One-way ANOVA statistical analysis was conducted (Dunnett's multiple comparisons test). Differences are compared to the control condition or between cell lines. p-values: 0.0332(*), 0.0021(**), 0.0002(***)

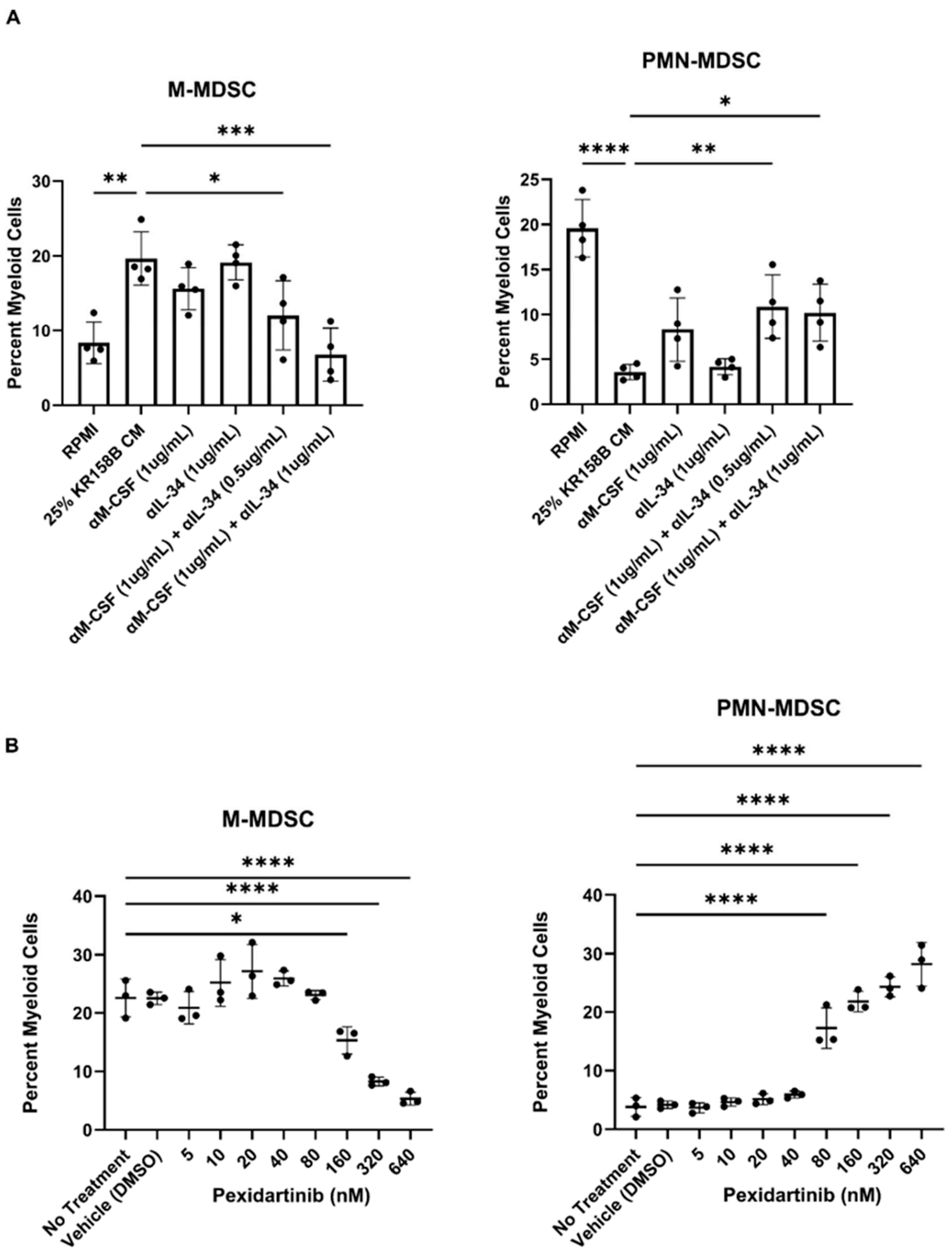

Figure S2. Inhibition of the CSF1R axis impacts percentage of M-MDSCs. (**A**) Whole bone marrow from wildtype mice were cultured in the presence of KR158B glioma conditioned media with M-CSF and IL-34 neutralizing antibody. M-MDSC percentage decreased to control levels with combination neutralization. PMN percentages were reduced in all conditions. (**B**) Whole bone marrow from wildtype mice was cultured in the presence of KR158B glioma conditioned media and the potent small molecule CSF1R inhibitor Pexidartinib. CSF1R inhibition resulted in a dose dependent decrease in M-MDSCs and an apparent increase in PMN-MDSCs percentage. One-way ANOVA

statistical analysis was conducted (Dunnett's multiple comparisons test). Differences are compared to the control condition or between cell lines. p-values: 0.0332(*), 0.0021(**), 0.0002(***).

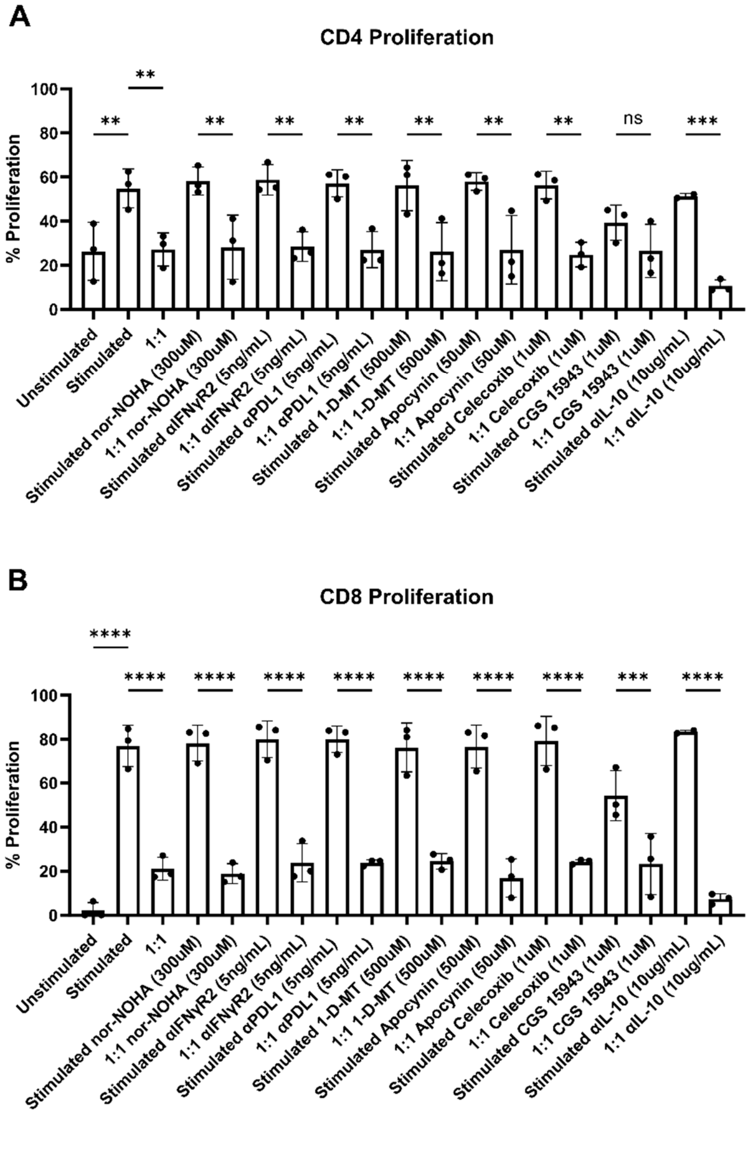

Figure S3. Inhibitor screen to identify mechanisms of glioma induced M-MDSC mediated T cell suppression. (**A**) Quantification of CD4 T cell proliferation in the presence of 1:1 ratio of M-MDSCs generated from KR158B conditioned media. Inhibitors targeting ARG1, IFNγR2, PDL1, IDO, NOX, COX2, A2AR, and IL-10 were tested to recover suppression induced by M-MDSCs. (**B**) Quantification of CD8 T cell proliferation. The tested inhibitors could not recover the proliferation of CD4 and CD8 T cells. One-way ANOVA statistical analysis was conducted (Dunnett's multiple comparisons test). Differences are compared to the stimulated control condition. p-values: 0.0332(*), 0.0021(**).

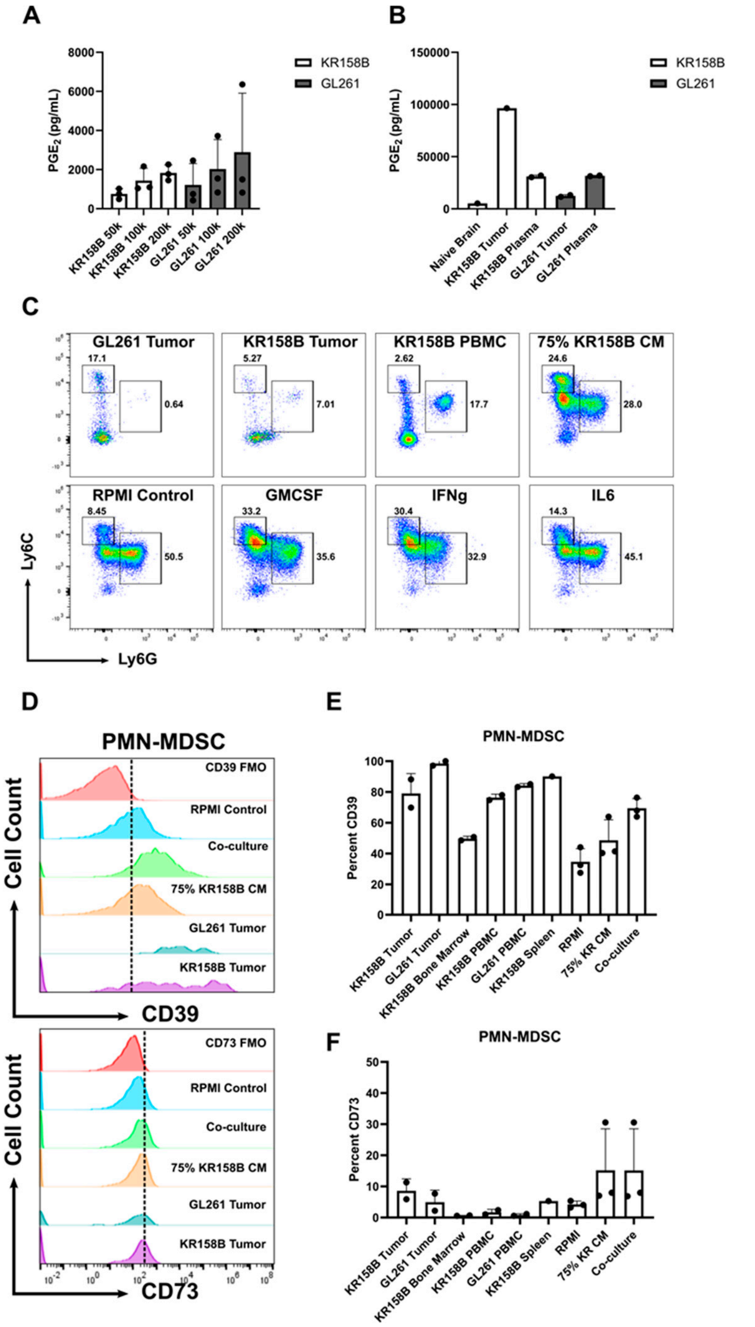

Figure S4. Glioma cells secrete PGE2 and PMN-MDSCs regulate CD39. (**A**) PGE2 ELISA on cultured GL261 or KR158B glioma conditioned media or tumors. (**B**) Representative flow cytometry analysis depicting gating strategy and percentage of M-MDSCs evaluated for CD39 and CD73 expression. (**C**) Representative flow cytometry histograms. (**E-F**) Graphs showing percentage of PMN-MDSCs expressing CD39 or CD73 on cell surface.

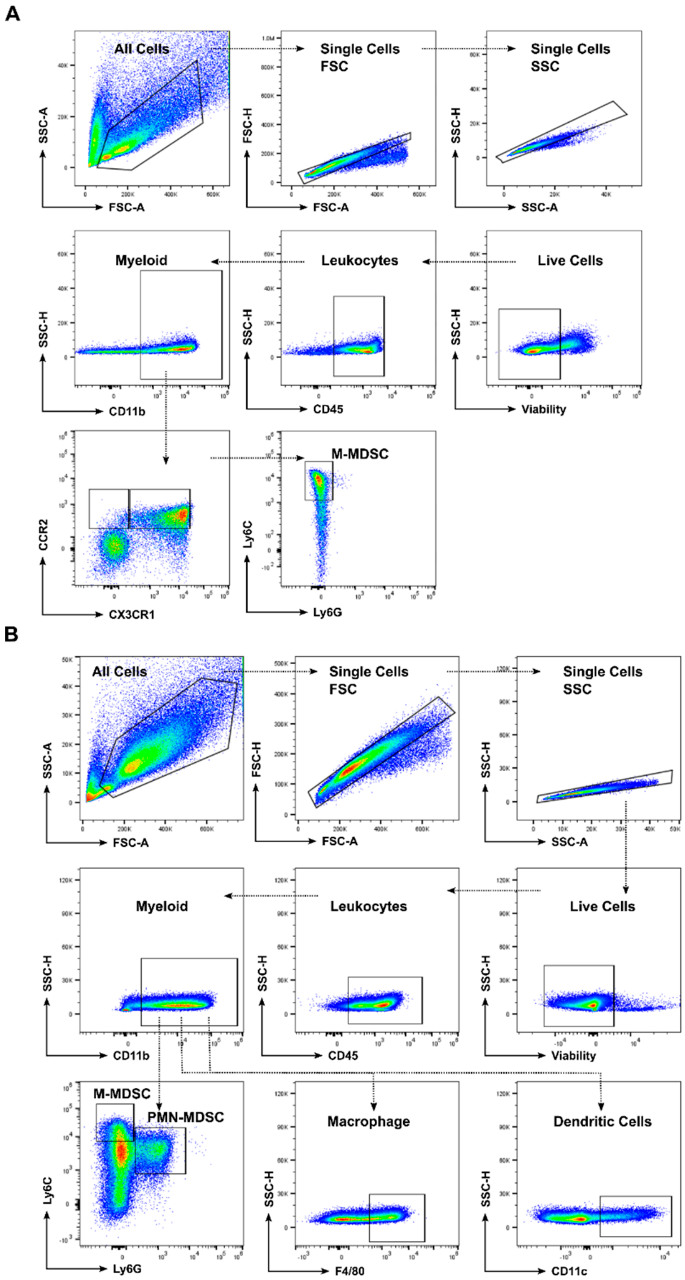

Figure S5. Representative flow cytometry gating strategy. (**A**) Flow cytometry gating strategy for CCR2/CX3CR1 expressing cells and CCR2/CX3CR1 expressing M-MDSCs generated from whole bone marrow cultured with KR158 conditioned media. Representative plot shows bone marrow treated with 75% KR158B conditioned media (**B**) Flow cytometry gating strategy for MDSC subsets, macrophages, and dendritic cells generated from whole bone marrow cultured with exogenous cytokines and KR158B conditioned media. Representative plot shows bone marrow treated with exogenous GM-CSF (40ng/mL).
